# Nasal sprays and a behavioural intervention for respiratory tract infections in primary care: 12-month follow-up of a randomised open-label trial

**DOI:** 10.3399/BJGP.2025.0269

**Published:** 2026-03-24

**Authors:** Paul Little, Jane Vennik, Kate Rumsby, Beth Stuart, Taeko Becque, Michael Moore, Nick Francis, Alastair D Hay, Theo Verheij, Katherine Bradbury, Kate Greenwell, Laura Dennison, Sian Holt, James Denison-Day, Ben Ainsworth, James Raftery, Tammy Thomas, Christopher C Butler, Samantha Richards-Hall, Debs Smith, Hazel Patel, Samantha Williams, Jane Barnett, Karen Middleton, Sascha Miller, Sophie Johnson, Jacqui Nuttall, Fran Webley, Tracey Sach, Lucy Yardley, Adam WA Geraghty

**Affiliations:** 1 Primary Care Research Centre, University of Southampton, Southampton, UK; 2 Wolfson Institute of Population Health, Faculty of Medicine and Dentistry, Queen Mary University of London, London, UK; 3 Centre for Academic Primary Care, Population Health Sciences, Bristol Medical School, University of Bristol, Bristol, UK; 4 Julius Center for Health Sciences and Primary Care, University Medical Center Utrecht, Utrecht, the Netherlands; 5 School of Psychology, University of Southampton, Southampton, UK; 6 Health Economics Analysis Team, University of Southampton, Southampton, UK; 7 Nuffield Department of Primary Care Health Sciences, University of Oxford, Oxford, UK; 8 Southampton Clinical Trials Unit, University of Southampton, Southampton, UK; 9 School of Psychological Science, University of Bristol, Bristol, UK

**Keywords:** behavioural support, general practice, nasal sprays, primary health care, respiratory illness

## Abstract

**Background:**

The Immune Defence trial documented a short-term impact on respiratory tract infections (RTIs) for nasal sprays and a stress management and physical activity website.

**Aim:**

To estimate the impact of sprays and the website after 12 months.

**Design and setting:**

A four-arm parallel randomised controlled trial. Participants with comorbidities and/or ≥3 self-reported recurrent RTIs were recruited.

**Method:**

Participants were randomised by online software (stratified by recurrent illness and comorbidities) to a) usual care (*n* = 3451); b) Vicks First Defence (VFD) spray (*n* = 3448) (two sprays/nostril, ≤6 times a day; c) isotonic saline spray (*n* = 3450) (same dosing); or d) a website promoting physical activity and stress management (*n* = 3450). The primary outcome was respiratory illness days.

**Results:**

Usual care participants (*n* = 3052) had on average 21.8 (standard deviation [SD] 35.2) illness days, reduced by VFD (*n* = 3076; 17.8 [SD 27.9] days, adjusted incidence rate ratio [IRR] 0.84, 99% confidence interval [CI] = 0.79 to 0.90, *P*<0.0001), and saline (*n* = 3142; 17.7 [SD 21.1 ] days, IRR 0.83, 99% CI = 0.78 to 0.89, *P*<0.0001), but not the website (*n* = 2811; 19.5 [SD 31.2] days, IRR 0.94, 99% CI = 0.88 to 1.01, *P* = 0.03). The website reduced incident infections (adjusted risk ratio [RR] 0.96, 95% CI = 0.93 to 0.99, *P* = 0.006). All interventions reduced symptom severity and work days lost, both spray groups reported lower intention to consult and fewer falls, and there were fewer antibiotic courses and practice visits with saline. Among those with recurrent illness, saline had the most impact on both recurrence and symptom days (RR 0.93, 95% CI = 0.87 to 0.99 and RR 0.70, 95% CI = 0.60 to 0.82, respectively). Headaches were higher for VFD and lower for saline (7.8% and 3.4%, respectively; 4.7% usual care).

**Conclusion:**

Widely available, inexpensive sprays and a website promoting self-care reduce the incidence, duration, and/or severity of RTIs and have an impact on work days lost and healthcare use.

## How this fits in

Limited prior evidence suggested that antiviral nasal sprays, or supporting physical activity and stress management, could lessen the impact of respiratory tract infections (RTIs), which drive the regular winter health crises. This evidence was supported by the 6-month results of the Immune Defence trial of a ‘free-standing’ website supporting physical activity and stress management, Vicks First Defence nasal spray, and a nasal saline spray, but the longer-term impacts are unknown. After 12 months, the website reduced incident infections by 4%. Both sprays resulted in four fewer illness days, fewer falls, and reported lower intention to consult. All interventions reduced symptom severity and work days lost. The saline group reported fewer practice visits and was most effective for those with recurrent illnesses.

## Introduction

Respiratory tract infections (RTIs) are one of the commonest reasons to attend primary care annually, and most attending GPs still prescribe antibiotics,^1,2^ which drives antibiotic resistance.^3^ Effective non-prescription interventions are needed to improve self-management of RTIs and limit the impact of the regular winter crises for the health service.

Some prior evidence suggested that sprays that used lower pH and a polymer^4–8^ could potentially reduce the viral load, and hence reduce the number and severity of illnesses.^7,9–11^ A systematic review of carrageenan sprays found an impact on symptom severity and possibly illness duration.^12^ There was also some evidence that saline alone might be effective;^11^ this was supported by observational evidence, evidence from a trial in children, and more recently both in vitro work (documenting the antiviral properties of saline) and a pilot study.^13–16^

Prior evidence also suggested an impact on symptom days with exercise and/or management of stress,^17–26^ but with relatively intensive interventions. Based on the accessibility and efficiencies of digital platforms, the authors of the current study developed a brief behavioural intervention (hosted on the ‘Immune Defence’ study website) based on modules of prior studies and requiring no support aiming to both increase physical activity^27–31^ (‘Getting Active’) and improve stress.^32^ The authors of the current study also developed modules to support using nasal sprays.

The primary analysis of the Immune Defence trial (*n* = 13 799) at 6 months demonstrated that the behavioural website reduced infection incidence (a relative reduction of 5%), and that both Vicks First Defence (VFD) nasal sprays and an isotonic saline spray reduced days of illness (a relative reduction of 20%). The longer-term impact of the interventions is unknown, hence in the current study the 12-month follow-up data from the trial are reported.

## Method

The study was prospectively registered on ISRCTN on 30 October 2020 (reference number: 17936080). Full details of the study methods are available in the 6-month published data.^33^

### Design summary

#### Invitation

Participants were invited from GP practices over 3 years from 12 December 2020 to 7 April 2023. Automated searches were used to identify potentially eligible patients.^33^

#### Inclusion criteria

Inclusion criteria were: aged ≥18 years; ≥1 comorbidity/risk factor(for example, heart disease; asthma/lung disease; diabetes), and/or ≥3 RTIs in a normal year.^33^

#### Exclusion criteria

Exclusion criteria included terminal illness or palliative care; residential care; dementia; pregnancy or breastfeeding; regularly using nasal sprays to prevent infections; and no internet access.^33^

#### Automated randomisation

The Immune Defence website software (designed by the Global Initiative company) randomised participants to the four trial groups, stratified by a) being in a higher risk group (aged >65 years and/or having comorbid condition); and/or b) recurrent illness: having had ≥3 RTIs in the last year.^33^

### Intervention groups

Intervention groups were as follows:^33^

- Usual care: brief advice for illness management based on current NHS advice.
- VFD nasal spray (which contains a polymer and buffers pH).
- Isotonic buffered saline (Sterinase) nasal spray.^11^
  In both spray groups, participants were asked to use the spray:
  - at first signs of an illness: two sprays in each nostril up to six times daily until symptom free;
  - after potential exposure to infection (such as, supermarkets and public transport): two sprays immediately after exposure in each nostril, an hour later and at the end of the evening; and
  - after prolonged exposure (such as, living with a person who has an infection): up to six times daily until recovery of the close contact.
    Both sprays were classed as medical devices. To reduce possible intervention contamination, both the nasal sprays were masked by overlabelling with generic study labels (‘gel-based nasal spray’ for VFD and ‘liquid-based nasal spray’ for saline). Sprays were resupplied on request. Participants were given online motivational information (brief content on the impact of RTIs and how nasal sprays can prevent RTIs) and instructions, supported by paper booklets, developed iteratively using the person-based approach.^34,35^
- Behavioural website promoting physical activity and stress management. This was also developed using the person-based approach.^34^ Participants had access to information on the impact of RTIs, how physical activity and/or stress management could prevent RTIs, and then two online modules supporting physical activity and stress reduction. Participants were also sent optional pedometers, to help personally monitor activity.

### Outcomes

Unless specified, outcomes were measured using a repeated questionnaire every 28 days for 12 months, and also at 6 and 12 months to recall infections since the start of the trial.

#### Primary outcome

The primary outcome was days of illness from self-reported RTIs (coughs, colds, sore throat, sinus or ear infections, and flu, including COVID-19).

Research has shown that people can remember the incidence and duration of illness over a few months,^10,36,37^ and in the PRIMIT trial^10^ and in the primary analysis of the Immune Defence trial^33^ estimates from self-report after several months were very similar to estimates from monthly reports.

#### Secondary outcomes

The secondary outcomes were incidence of illness (in both contemporaneous monthly questionnaires and retrospectively at 6 months);^10^ possible harms; days with symptoms moderately bad or worse;^37,38^ days where work/normal activities were impaired; use of antibiotics;^36^ health service contacts;^38^ number of days of respiratory illness over 12 months;^10^ and at 6 and 12 months: self-reported belief in the effectiveness of antibiotics (Likert scale); intention to consult with similar illnesses for future episodes (Likert scale); and mental health (using the Perceived Stress Scale,^39^ Patient Health Questionnaire [PHQ]-8,^40^ and Generalised Anxiety Disorder [GAD]-7).^41^

Other secondary outcomes to be reported subsequently were:

- NHS contacts through participant self-report and retrospective notes review;
- health-related quality of life elicited using the five-level EQ-5D; and
- engagement with the trial interventions, evaluated through participant self-report, and usage data from the trial website.

### Data collection

Unless specified, data were collected using the trial website designed by Global Initiative blind to group, with up to two email reminders, then a mailed questionnaire, and finally a telephone call as necessary by blinded members of the study team for individuals who had not completed data for the primary outcome. Data from paper questionnaires and telephone interviews were entered into a secure access database by the trial team.

### Sample size calculations

Sample size calculations were based on data of the incidence of illness (blind to group) from the first two seasons (2020/2021 and 2021/2022):

- Stratum 1 (recurrence, no risk factors): 71% (not 15% as was assumed) had an illness. Using the lower limit of the 95% confidence interval (CI) of this estimate it was assumed at least 65% would get an illness. Based on the original 147 per group (that is, changing no other assumptions), it was estimated 226 per group were needed, and 1130 total (with 80% follow-up).
- Stratum 2 (risk factors, no recurrence): 40% had illness (not 15% as was assumed), requiring 147/0.4 = 368 per group, and 1472 in total.
- Stratum 3 (risk factors plus recurrence): 62% had illness, requiring 245 per group, and 1225 in total.

### Data analysis

A detailed statistical analysis plan was finalised before data analysis and data lock, and superseded the brief protocol description. Count outcomes, including the number of days of illness, were analysed using zero-inflated negative binomial regression models given the large number of zeros because of no illness. Logistic regression was used for dichotomous outcomes and linear regression for continuous outcomes. Skewed outcomes were either transformed before linear regression or analysed using Poisson regression with robust standard errors. All models adjusted for baseline days of illness and stratification variables. Multiple imputation with chained equations was used for the incidence of illness, using 100 imputations.^42^

The imputation model included all variables in the analysis model (that is, outcome, baseline days, and strata) and variables that predicted missingness (age, sex, Index of Multiple Deprivation decile, baseline belief in antibiotics, and baseline intention to consult). For the primary outcome, it was assumed the data were missing completely at random, but sensitivity analyses for this assumption were given where missing data were imputed as either 0 or 30 days.^43^

### Patient and public involvement

Three public contributors helped develop the protocol and all materials (for example, patient information and topic guides), contributed to management regarding development/operationalising the study, and with outputs (such as lay summaries and publications).

## Results

### Recruitment and follow-up

**Figure 1.**
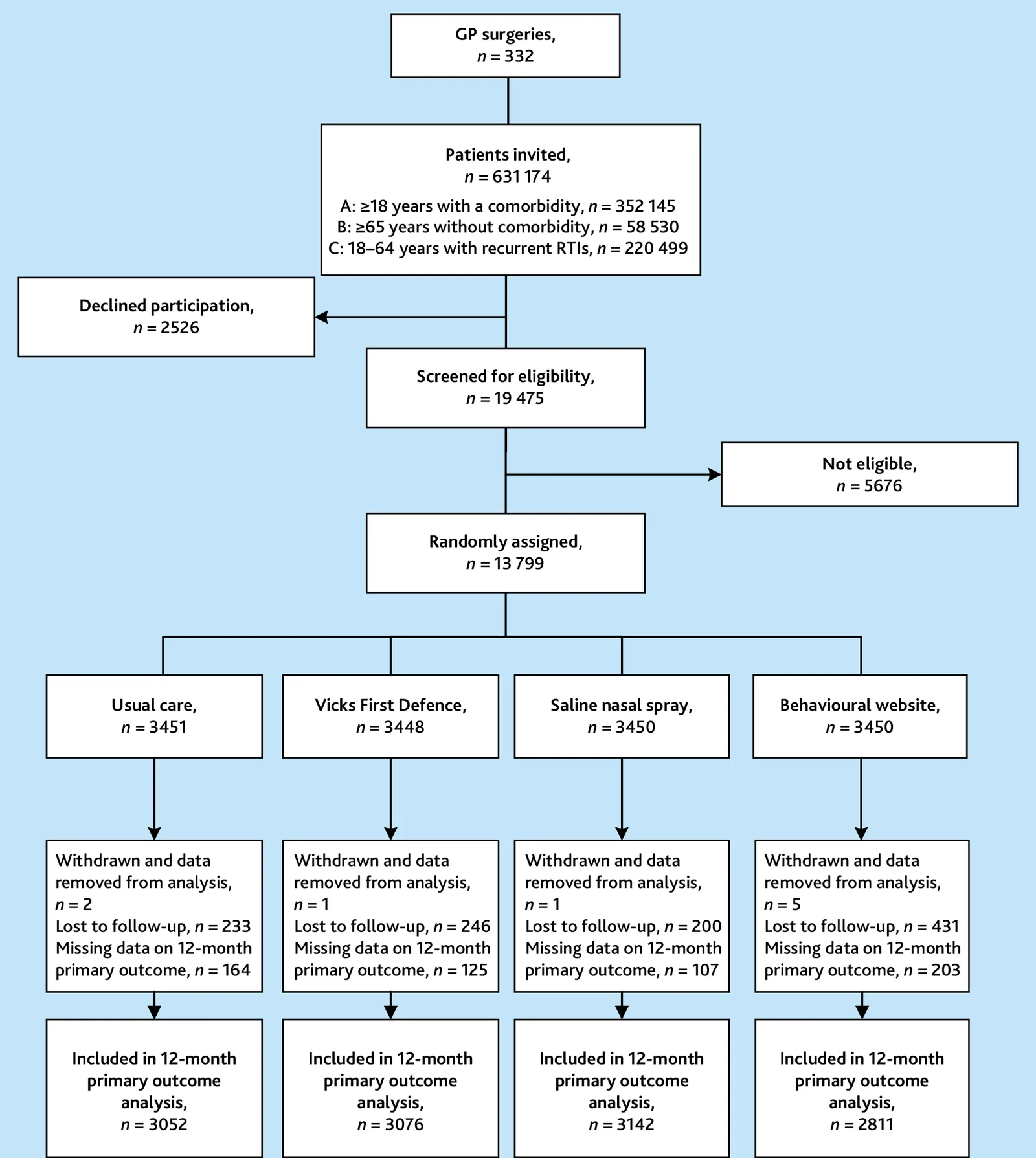
CONSORT diagram.

In total, 13 799 participants were recruited in three winter seasons (September to April) from 12 December 2020 to 7 April 2023 (Figure 1). Groups were well balanced (Table 1), and follow-up was good based on monthly data (usual care, *n* = 3052/3449, 88.5%; VFD, *n* = 3076/3447, 89.2%; saline, *n* = 3142/3449, 91.1%; and behavioural website, *n* = 2811/3445, 81.6%). The monthly reports of days of illness were spread throughout the 12-month period.

**Table 1.** Baseline characteristics of the randomised population

Characteristic^a^	Randomised group			
	Usual care,*n* = 3451	Vicks First Defence,*n* = 3448	Saline,*n* = 3450	Behavioural website,*n* = 3450
**Sex**				
Male	1548 (44.9)	1536 (44.6)	1488 (43.2)	1526 (44.3)
Female	1890 (54.8)	1900 (55.2)	1953 (56.7)	1904 (55.3)
Other	5 (0.1)	4 (0.1)	2 (0.1)	10 (0.3)
Prefer not to say	3 (0.1)	5 (0.1)	4 (0.1)	4 (0.1)
Missing	5	3	3	6
**Age, median (IQR)**	64 (51–71)	65 (50–71)	64 (50–71)	64 (51–71)
Missing	2	1	1	5
**Ethnicity**				
White	3328 (97.1)	3324 (97.0)	3334 (97.1)	3319 (96.8)
Mixed	25 (0.7)	35 (1.0)	28 (0.8)	38 (1.1)
Asian	51 (1.5)	49 (1.4)	46 (1.3)	54 (1.6)
Black	16 (0.5)	11 (0.3)	12 (0.3)	6 (0.2)
Other	9 (0.3)	9 (0.3)	14 (0.4)	11 (0.3)
Missing	22	20	16	22
**Marital status**				
Single	402 (11.7)	398 (11.6)	424 (12.3)	428 (12.5)
Married	2474 (71.9)	2439 (71.0)	2423 (70.5)	2408 (70.1)
Widowed	216 (6.3)	225 (6.5)	229 (6.7)	222 (6.5)
Divorced	292 (8.5)	320 (9.3)	303 (8.8)	308 (9.0)
Separated	55 (1.6)	54 (1.6)	59 (1.7)	71 (2.1)
Missing	12	12	12	13
**Education**				
No qualifications	196 (5.7)	188 (5.5)	212 (6.2)	216 (6.3)
GCSE	711 (20.7)	749 (21.8)	730 (21.2)	694 (20.2)
A-level	588 (17.1)	572 (16.6)	568 (16.5)	587 (17.1)
HNC/HND	288 (8.4)	289 (8.4)	283 (8.2)	325 (9.4)
Degree	898 (26.1)	891 (25.9)	893 (26.0)	872 (25.3)
Higher degree	204 (5.9)	247 (7.2)	216 (6.3)	239 (6.9)
Postgraduate	409 (11.9)	380 (11.1)	401 (11.7)	392 (11.4)
Other	146 (4.2)	121 (3.5)	137 (4.0)	117 (3.4)
Missing	11	11	10	8
**Number in household, median (IQR)**	2 (2–3)	2 (2–3)	2 (2–3)	2 (2–3)
Missing	23	29	13	38
**Children aged <16 years in household**	545 (16.0)	514 (15.1)	531 (15.6)	483 (14.2)
Missing	43	52	41	46
**BMI, mean (SD)**	28.6 (6.9)	28.5 (8.1)	28.5 (6.9)	28.4 (6.9)
Missing	80	82	56	71
**Current smoker**	178 (5.2)	192 (5.6)	177 (5.2)	157 (4.6)
Missing	49	34	40	36
**Any comorbidity**	2724 (79.0)	2706 (78.5)	2699 (78.3)	2687 (78.0)
Missing	2	1	1	5
**Number comorbidities, median (IQR)**	1 (1–2)	1 (1–2)	1 (1–2)	1 (1–2)
Flu vaccination in last 12 months	2912 (85.0)	2888 (84.6)	2882 (84.2)	2907 (85.0)
Missing	26	36	27	31
COVID vaccination in last 12 months	3045 (88.3)	3019 (87.6)	3040 (88.1)	3000 (87.1)
Missing	2	1	1	5
**COVID illness in last 12 months**				
Yes	1068 (31.2)	1070 (31.2)	1034 (30.1)	1038 (30.2)
No	2253 (65.8)	2236 (65.2)	2290 (66.6)	2277 (66.3)
Not sure	103 (3.0)	123 (3.6)	116 (3.4)	117 (3.4)
Missing	27	19	10	18
**Days of COVID symptoms**	1032	971	1017	997
Median (IQR)	8 (5–14)	8 (5–14)	7 (5–12)	8 (5–14)
**Previous use nasal spray**	2826 (85.3)	2800 (84.5)	2811 (84.8)	2846 (85.9)
Missing	138	135	137	138
**Had RTI in a normal year**	3093 (89.7)	3106 (90.1)	3065 (88.9)	3114 (90.4)
Missing	2	1	1	5
**Number RTIs in a normal year**	3054	3073	3039	3076
Median (IQR)	2 (1–3)	2 (1–3)	2 (1–3)	2 (1–3)

^a^ *n *(%), unless otherwise stated. HNC = higher national certificate. HND = higher national diploma. IQR = interquartile range. RTI = respiratory tract infection.

There were 10 participants who asked for their data to be removed, and 679 withdrew but allowed data use, leaving 13 789 participants: stratum 1 (recurrence, no risk factors), *n* = 1217 (8.8%); stratum 2 (risk factors, no recurrence), *n* = 8652 (62.7%); and stratum 3 (risk factors plus recurrence), *n* = 3920 (28.4%).

Reasons for withdrawal provided were: unable to engage with intervention (*n* = 116: behavioural website, *n* = 53, 45.7%; VFD *n* = 27, 23.3%; saline, *n* = 27, 23.3%; and usual care, *n* = 9, 7.8%); too unwell/change in medical condition (*n* = 109); trial processes/too busy (*n* = 107); personal circumstances (*n* = 45); deceased (*n* = 42); study not relevant/does not get RTIs (*n* = 28); other (*n* = 14); pregnancy/breastfeeding (*n* = 3); and no reason given (*n* = 215).

### Days with illness

Asking about infections since the beginning of the trial (12 months) was apparently interpreted by some participants for the last 6 months (probably because they were asked about infections for the first 6 months, and assumed the same time period applied at the 12-month follow-up) — with participants reporting much lower rate of infections than was plausible (for example, the saline group, where reported days of illness [a mean of 6 days] was lower than at the 6-month time point). The monthly data were therefore used in the current study as providing the more reliable data for the primary outcome, supported by the estimates for complete 12-month data that were very similar to those where at least 1 month was recorded. Using the latter data, usual care participants (*n* = 3052) had on average 21.8 (standard deviation [SD] 35.2) illness days, reduced by VFD (*n* = 3076; 17.8 [SD 27.9] days, adjusted incidence rate ratio [IRR] 0.84, 99% CI = 0.79 to 0.90; *P*<0.0001) and saline (*n* = 3142; 17.7 [SD 21.1] days, IRR 0.83, 99% CI = 0.78 to 0.89; *P*<0.0001), but not the website (*n* = 2811; 19.5 [SD 31.2] days, IRR 0.94, 99% CI = 0.88 to 1.01; *P* = 0.03) (Table 2).

**Table 2.** Days of illness using data every 28 days summed over 12 months (that is, recall period 28 days)

Category	Randomised group			
	Usual care, *n =* 3449	Vicks First Defence,*n* = 3447	Nasal saline,*n* = 3449	Behavioural website,*n* = 3445
**Days of illness (participants with all 12 months’ monthly data)**				
*n*	1548	1531	1564	1269
Median (IQR)	15 (5–32)	13 (4–29)	14 (5–28)	14 (4–29)
Mean (SD)	24.3 (33.6)	20.7 (23.6)	20.9 (24.1)	23.1 (34.9)
Adjusted IRR (99% CI)	Reference	**0.83 (0.76 to 0.91)**	**0.85 (0.77 to 0.92)**	0.94 (0.85 to 1.03)
**Days of illness (participants with ≥1 month of data** ^**a**^ **)**				
*n*	3052	3076	3142	2811
Median (IQR)	13 (3–29)	10 (2–24)	11 (3–25)	10 (0–25)
Mean (SD)	21.8 (35.2)	17.8 (27.9)	17.7 (22.1)	19.5 (31.2)
Adjusted IRR (99% CI)	Reference	**0.84 (0.79 to 0.90)**	**0.83 (0.78 to 0.89)**	0.94 (0.88 to 1.01)

a Assuming missing monthly days of illness are zero. Bold indicates statistically significant at 5% level. IQR = interquartile range. IRR = incidence rate ratio. SD = standard deviation.

### Secondary outcomes

Occurrence of infections was lower in the behavioural website group (adjusted risk ratio [RR] 0.96, 95% CI = 0.93 to 0.99), but not the spray groups (Table 3).

**Table 3.** Key secondary outcomes — occurrence of infection over the last 12 months^a^

Category	Usual care,*n* = 3449	Vicks First Defence,*n* = 3447	Nasal saline,*n* = 3449	Behavioural website,*n* = 3445
**Occurrence of infection in last 12 months**				
Reported RTI, *n*/*N* (%)	1923/2612 (73.6)	1887/2503 (75.4)	1917/2534 (75.7)	1643/2320 (70.8)
Missing, *n* (%)	837 (24.3)	944 (27.4)	915 (26.5)	1125 (32.7)
Adjusted^b^ RR (95% CI)	Reference	1.01 (0.98 to 1.03)	1.01 (0.98 to 1.03)	**0.96 (0.93 to 0.99)**
**Infections in last 12 months**				
*n*	2584	2488	2522	2297
Median (IQR)	1 (0–3)	2 (1–3)	2 (1–3)	1 (0–2)
Mean (SD)	1.94 (2.27)	1.87 (1.94)	1.86 (1.94)	1.80 (2.18)
Adjusted IRR (95% CI)^b,c^	Reference	0.94 (0.89 to 1.01)	0.94 (0.88 to 1.00)	0.95 (0.88 to 1.02)

^a^Complete cases analysis. ^b^IRR for intervention versus usual care. ^c^Adjusted for baseline number of days of RTI symptoms and stratum; bold indicates statistically significant at 5% level. IQR = interquartile range. IRR = incidence rate ratio. RR = risk ratio. RTI = respiratory tract infection. SD = standard deviation.

Moderately bad symptoms were significantly reduced in all intervention groups as were days of work lost. Participants in the saline group, but not the other intervention groups, reported significantly fewer visits to a healthcare professional for an RTI (IRR 0.81, 95% CI = 0.68 to 0.97) and fewer antibiotic courses (IRR 0.84, 95% CI = 0.71 to 1.00) (Table 4).

**Table 4.** Secondary outcomes — 12 months^a^

Category	Usual care,*n* = 3449	Vicks First Defence,*n* = 3447	Nasal saline,*n* = 3449	Behavioural website,*n* = 3445
**Days of moderately bad symptoms over last 12 months,** ***n***	2605	2501	2529	2310
Median (IQR)	2 (0–5)	2 (0–5)	2 (0–5)	2 (0–5)
Mean (SD)	4.9 (10.0)	4.1 (8.7)	3.9 (7.1)	4.1 (7.8)
IRR (95% CI)	Reference	**0.86 (0.80 to 0.94)**	**0.83 (0.76 to 0.90)**	**0.91 (0.83 to 0.99)**
**Days of work lost over last 12 months, *n***	2575	2465	2510	2273
Median (IQR)	0 (0–3)	0 (0–2)	0 (0–2)	0 (0–2)
Mean (SD)	3.0 (8.4)	2.3 (6.9)	2.1 (5.3)	2.2 (5.4)
IRR (95% CI)	Reference	**0.84 (0.74 to 0.97)**	**0.82 (0.72 to 0.94)**	**0.85 (0.74 to 0.97)**
**Times saw HCP for an RTI over last 12 months, *n***	2609	2500	2531	2314
Median (IQR)	0 (0–0)	0 (0–0)	0 (0–0)	0 (0–0)
Mean (SD)	0.44 (1.52)	0.33 (1.08)	0.32 (0.98)	0.37 (1.15)
IRR (95% CI)	Reference	0.88 (0.74 to 1.06)	**0.81 (0.68 to 0.97)**	1.01 (0.84 to 1.21)
**Courses of antibiotics over last 12 months, *n***	2580	2468	2513	2270
Mean (SD)	0.32 (0.96)	0.27 (0.86)	0.27 (0.78)	0.31 (0.99)
IRR (95% CI)	Reference	0.90 (0.75 to 1.07)	0.84 (0.71 to 1.00)	1.09 (0.91 to 1.30)
**Had COVID-19 over last 12 months,** ***n/N*** **(%)**	945/2569 (36.8)	865/2451 (35.3)	903/2499 (36.1)	817/2261 (36.1)
RR (95% CI)	Reference	0.96 (0.89 to 1.03)	0.98 (0.91 to 1.05)	0.99 (0.92 to 1.07)
**Belief in antibiotics, *n***	2492	2379	2448	2199
Mean (SD)	3.5 (1.6)	3.4 (1.5)	3.4 (1.6)	3.5 (1.6)
OR (95% CI)^b^	Reference	0.93 (0.83 to 1.02)	0.96 (0.87 to 1.06)	1.09 (0.98 to 1.21)
**Likelihood of seeing doctor for next infection, *n***	2509	2404	2452	2211
Mean (SD)	2.9 (1.4)	2.8 (1.3)	2.8 (1.3)	2.9 (1.4)
Mean diff (95% CI)	Reference	**−0.10 (−0.18 to −0.02)**	**−0.08 (−0.16 to −0.003)**	−0.04 (−0.12 to 0.04)
**Total IPAQ score at 12 months, *n***	2544	2426	2485	2231
Mean (SD)	2675 (2887)	2646 (2672)	2674 (2699)	2783 (2815)
IRR (95% CI)^c^	Reference	1.01 (0.96 to 1.06)	0.99 (0.94 to 1.04)	1.03 (0.98 to 1.08)
**Sitting hours weekday, *n***	2355	2283	2324	2032
Mean (SD)	8.7 (5.1)	8.7 (5.0)	8.9 (5.1)	8.6 (5.1)
Mean diff (95% CI)	Reference	0.04 (−0.21 to 0.29)	0.14 (−0.11 to 0.39)	−0.02 (−0.28 to 0.24)
**Perceived Stress Scale at 12 months, *n***	2301	2202	2246	2035
Mean (SD)	19.9 (8.9)	19.4 (8.7)	19.3 (8.7)	19.4 (9.2)
Mean diff (95% CI)	Reference	**−0.7 (−1.0 to −0.3)**	**−0.5 (−0.9 to −0.1)**	−0.3 (−0.7 to 0.04)
PHQ-8 at 12 months, *n*	2393	2280	2312	2090
Mean (SD)	4.4 (4.8)	3.9 (4.4)	3.9 (4.4)	3.9 (4.6)
Mean diff (95% CI)	Reference	**−0.08 (−0.13 to −0.02)**	**−0.06 (−0.11 to −0.00)**	**−0.07 (−0.12 to −0.02)**
GAD-7 at 12 months, *n*	219	159	172	2101
Mean (SD)	4.3 (4.7)	4.8 (5.2)	3.8 (4.2)	3.3 (4.3)
Mean diff (95% CI)	Reference	−0.06 (−0.72 to 0.60)	−0.27 (−0.92 to 0.39)	**−0.66 (−1.12 to −0.20)**

^a^Adjusted for baseline outcome and stratum. Bold indicates statistically significant at 5% level. ^b^OR from ordinal logistic regression. ^c^IRR from Poisson with robust standard errors. diff = difference. GAD = Generalised Anxiety Disorder. HCP = healthcare professional. IPAQ = International Physical Activity Questionnaire. IQR = interquartile range. IRR = incidence rate ratio. OR = odds ratio. PHQ = Patient Health Questionnaire. RR = risk ratio. SD = standard deviation.

Both spray groups were less likely to intend seeking care for subsequent infections, and had significantly lower scores for perceived stress and depression (PHQ-8). Anxiety (GAD-7 score) was significantly lower in the website group compared with the usual care group (Table 4).

The results in each stratum suggest among those with recurrent illness saline had the most impact on both recurrence and symptom days (RR 0.93, 95% CI = 0.87 to 0.99 and RR 0.70, 95% CI = 0.60 to 0.82, respectively) whereas the behavioural website was most effective among those with comorbidities (Supplementary Table S1).

### Use of sprays

Participants were supplied with two bottles of nasal spray by post as soon as possible after randomisation. There were 1864 people who requested additional sprays (*n* = 937 saline and *n* = 927 VFD) during their participation. The mean number of additional bottles supplied per individual were 1.66 (range 1–18, SD 1.27) for saline and 1.69 (range 1–15, SD 1.35) for VFD (data not shown).

### Adverse events

Headache was more common with VFD and less common for saline, but both spray groups had less nasal dryness and irritation, and fewer reported falls (Table 5).

**Table 5.** Adverse events of special interest — 12 months^a^

Adverse event, *n* (%)^a,b^	Usual care	Vicks First Defence	Nasal saline	Behavioural website
**Headache/sinus pain**	2537	2408	2468	2223
Yes	120 (4.7)	187 (7.8)	85 (3.4)	110 (4.9)
No	2344 (92.4)	2136 (88.7)	2330 (94.4)	2070 (93.1)
Not sure	73 (2.9)	85 (3.5)	53 (2.1)	43 (1.9)
RR (95% CI)	Reference	**1.61 (1.29 to 2.00)**	**0.73 (0.56 to 0.95)**	1.05 (0.82 to 1.35)
**Heavy nosebleed**	2535	2407	2464	2218
Yes	72 (2.8)	85 (3.5)	77 (3.1)	83 (3.7)
No	2449 (96.6)	2309 (95.9)	2377 (96.5)	2126 (95.9)
Not sure	14 (0.6)	13 (0.5)	10 (0.4)	9 (0.4)
RR (95% CI)	Reference	1.23 (0.91 to 1.69)	1.10 (0.81 to 1.51)	1.32 (0.97 to 1.80)
**Nasal dryness/irritation**	2538	2409	2469	2223
Yes	703 (27.7)	625 (25.9)	513 (20.8)	577 (26.0)
No	1740 (68.6)	1709 (70.9)	1875 (75.9)	1558 (70.1)
Not sure	95 (3.7)	75 (3.1)	81 (3.3)	88 (4.0)
RR (95% CI)	Reference	**0.91 (0.83 to 0.99)**	**0.73 (0.67 to 0.81)**	0.93 (0.84 to 1.02)
**Slight nosebleed**	2536	2406	2469	2225
Yes	407 (16.0)	367 (15.3)	357 (14.5)	342 (15.4)
No	2098 (82.7)	2008 (83.5)	2083 (84.4)	1861 (83.6)
Not sure	31 (1.2)	31 (1.3)	29 (1.2)	22 (1.0)
RR (95% CI)	Reference	0.95 (0.83 to 1.08)	0.90 (0.79 to 1.02)	0.96 (0.84 to 1.10)
**Trips/falls**	2537	2408	2468	2226
Yes	586 (23.1)	414 (17.2)	420 (17.0)	475 (21.3)
No	1915 (75.5)	1968 (81.7)	2014 (81.6)	1720 (77.3)
Not sure	36 (1.4)	26 (1.1)	34 (1.4)	31 (1.4)
RR (95% CI)	Reference	**0.74 (0.66 to 0.83)**	**0.73 (0.66 to 0.82)**	0.92 (0.83 to 1.02)

^a^RR assuming ‘not sure’ categorised as ‘no’. Adjusted for baseline days of illness and stratum. Bold indicates statistically significant at 5% level. ^b^Unless otherwise stated. RR = risk ratio.

## Discussion

### Summary

This trial provides good evidence for the longer-term impact of robustly developed^35,44^ accessible, easily scalable interventions for preventive or very early use to reduce symptom days of RTIs in primary care. Both nasal sprays reduced the number of days of illness over a year from 22 to 18 days. All three interventions also reduced the severity of symptoms and work days lost. Illness incidence was also reduced by the behavioural website.

### Strengths and limitations

The study was open label, but since the mechanisms of action of sprays are complex (probably a mixture of washing out virus and an antiviral effect)^14^ devising a meaningful placebo would be difficult, and nasal sprays were relabelled (retaining some blinding). Placebo effects and resentful demoralisation^45^ in open trials could bias results, but for RTIs the estimates from open-label trials (for example, sore throat,^36^ acute bronchitis,^37^ and otitis^46^— where placebo effects and resentful demoralisation are possible) are very similar to placebo controlled trials^38,47–49^ (where resentful demoralisation is very unlikely — including trials of medicines in COVID-19).^50,51^ The impact on severity and work days lost, and the different pattern of impact for the website and the sprays also suggest non-specific ‘placebo’ effects are less likely.

Self-reported outcomes are not ‘objective’, but medical history examination and self-report agree moderately well;^52^ there is no meaningful alternative to self-report to assess symptom presence and severity, and self-report of normal activities and symptoms are sensitive to change and reliable.^36,53^ Reports of symptom duration after several weeks are reliable,^36,53^ and the reports over months are also comparable with monthly reports^10^ (presumably as relatively few infections makes remembering easier). The current study found evidence that some participants had misunderstood the timeframe of the questions for days of illness at 12 months, so for the days of illness the monthly data had to be used. The results in season 3 (post-pandemic) were slightly better than the pandemic period, and since SARS-CoV-2 remains prevalent throughout the year with coinfections common,^54^ the post-pandemic spike in infections makes the current results even more relevant for patients.

Although ‘cold calling’ mailed invitations provide low uptake rates, the PRIMIT trial (which used similar invitation methods)^10^ demonstrated comparable behavioural intentions outside the trial setting,^55^ which suggests the results may be generalisable. The novel methodology of recruitment via the internet and central distribution/supply of the sprays was very efficient and convenient for patients, reducing barriers to participation. There was clear evidence of suboptimal adherence to sprays (which the study reported in the first 6 months), particularly for preventive use of sprays.^33^ Despite slightly fewer participants from ethnic minorities (3.2% versus 5% 2021 census data for this age group), and more with qualifications above A-level (52% versus 34% census data), there was no clear effect of these imbalances on the estimates.

It is likely that the current study underestimated the impact of saline or VFD for several reasons: sprays were only resupplied on request and the authors know adherence to the recommended use of sprays was limited (particularly for preventive behaviours). The study also used isotonic saline rather than hypertonic saline, which is likely to be more effective given the mechanism of action of saline almost certainly involves the supply of chloride ions to cells.^13,14^

### Comparison with existing literature

All interventions largely retained the impact on the primary and secondary outcomes after 12 months to the impact at 6 months despite no routine resupply of sprays, albeit there was perhaps slightly less impact of sprays on days of illness (IRR 0.83 at 12 months versus IRR 0.80 at 6 months^33^) and antibiotic use. The impact of sprays is supported by smaller studies — by previous in vitro pilot work,^13,14^ a trial of saline in children and observational data,^15,16^ and trials of other nasal sprays.^7,9–11,56^ The reduction in illness severity and work days lost for all interventions was consistent with the 6-month findings, as was the small but important population effect of the behavioural website on infection incidence.^33^

The results for individual strata suggest that saline was most effective among participants with a history of recurrent illnesses. The VFD group reported more headache, and the saline group less headache when compared with usual care. An interesting finding was the reduction in self-reported falls in both spray groups. Despite concerns about measurement error in recall of falls,^57^ measurement error would be expected to make it more difficult to detect an effect. Furthermore, these findings are unlikely to be owing to chance given the finding was consistent in both spray groups — and if a real effect, it is presumably owing to lessening the impact of infections on balance: falls are known to be associated with infections in more frail individuals,^58,59^ and so the observed reduction in illness duration and severity might be expected to reduce falls. The experience of using the sprays might also reduce fear of falling, which is known to be a strong predictor of falls.^60,61^

The Cochrane review assessing the impact of physical activity on RTIs suggested promising effects of physical activity for symptom days;^17^ however, all the trials required intensive input. In the current study, the impact on symptom days was close to significance, similar to the estimate (2 days’ benefit) from the Cochrane review,^17^ and consistent with the effect of the intervention on more severe symptoms and work days lost. To the authors’ knowledge, the brief, unsupervised digital approach website is the first effective robustly developed, pragmatic, scalable intervention to support physical activity and stress management for preventing and managing RTIs.^35,44^

### Implications for research and practice

Advice to use inexpensive and widely available nasal sprays, even when not routinely resupplying them, has moderate but an important longer-term impact on a range of outcomes — illness duration, the severity of symptoms, work days lost, intention to consult health professional in future episodes, and the incidence of falls. Both sprays were well tolerated, albeit VFD was associated with slightly more headaches, and saline fewer headaches. Providing a physical activity and stress management website also retained a significant impact at 12 months for the incidence of illness, illness severity, and work days lost. The potential for sprays to prevent illness has not been tested adequately given the limited adherence to preventive use of sprays. Further research should concentrate on boosting adherence, and using hypertonic rather than isotonic saline.

## Open access

This article is Open Access: CC BY 4.0 licence (http://creativecommons.org/ licences/by/4.0/).

## Data

Data are available on request from Adam WA Geraghty or Paul Little with a proposal for further analysis.
